# Macular Thickness, Foveal Volume, and Choroidal Thickness in Amblyopic Eyes and Their Relationships to the Treatment Outcome

**DOI:** 10.1155/2018/1967621

**Published:** 2018-08-06

**Authors:** Chun-Hsiu Liu, Sherine Jue Ong, Chung-Ying Huang, Wei-Chi Wu, Ling-Yuh Kao, Meng-Ling Yang

**Affiliations:** ^1^Department of Ophthalmology, Chang Gung Memorial Hospital, Chang Gung University College of Medicine, No. 5, Fuhsing Street, Kweishan, Taoyuan 333, Taiwan; ^2^Ching Ming Eye Clinic, No. 32, Zhongshan 2nd Rd., Luzhou, New Taipei 247, Taiwan

## Abstract

**Purpose:**

To assess the correlations between the retinal/choroidal structure and the treatment outcomes of amblyopic children.

**Methods:**

This study enrolled eyes with amblyopia resulting from strabismus, anisometropia, or ametropia. All patients underwent detailed eye examinations, including spectral domain optical coherence tomography (SD-OCT) scan. All of the subjects received amblyopic treatment and were divided into 2 groups after 6 months of follow-up: the recovered amblyopic group with a best-corrected visual acuity (BCVA) ≥0.8 and the persistent amblyopic group with a BCVA <0.8 on the Landolt C chart.

**Results:**

Forty-four amblyopic children were included, of which 26 were in the recovered amblyopic group after 6 months of follow-up. The patients with strabismic anisometropic amblyopia and severe amblyopia (initial VA ≤ 0.3) were significantly predisposed to developing persistent amblyopia (*P*=0.049 and *P* < 0.001, respectively). After correcting with Littmann's formula, the thickness and volume of the parafoveal and perifoveal retinal regions in the persistent amblyopia group did not show significant differences with the recovered amblyopia group.

**Conclusions:**

The initial severity of amblyopia and the type of amblyopia were the risk factors related to the poor outcome of amblyopic treatment. The foveal thickness, foveal volume, and choroidal thickness were not associated with the treatment outcome.

## 1. Introduction

Amblyopia is the reduced best-corrected visual acuity (BCVA) of one eye or, less commonly, both eyes, which is caused by abnormal binocular interaction and the lack of adequate visual stimulation during visual development. The causes of amblyopia include strabismus, anisometropia, vision deprivation, or a combination of these factors [1]. Several morphological and functional changes have been observed in the lateral geniculate nucleus and visual cortex in subjects with various types of amblyopia [1–3].

Risk factors for the failure of amblyopia treatment have been evaluated in several reports, only a few of which discussed the correlation between the macular/choroidal biometric values and the results of treatment, and the relationship has not been clarified [4–6].

Optical coherence tomography (OCT) provides a highly qualitative, objective, and reproducible structural assessment of retinal structures. The OCT has evolved rapidly in the past few years, from time domain OCT to spectral domain OCT (SD-OCT) which allows more rapid scanning and higher resolution and permits a more detailed analysis of the optic nerve and retina. The application of OCT in amblyopia children has been demonstrated in several studies but with inconsistent results [6–9].

The purpose of this study was to evaluate the macular and choroidal thicknesses in persistent and recovered amblyopic children using SD-OCT. We sought to determine if any change in the macular or choroidal biometric values is related to the treatment outcome.

## 2. Materials and Methods

This study was performed at the Chang Gung Memorial Hospital between January 2012 and April 2013. The inclusion criteria included patients being older than 5 years and having a diagnosis of amblyopia, which was defined as BCVA less than 0.8. Severe amblyopia was defined as a BCVA of 0.3 or worse. The study was approved by the Ethics Committee of Chang Gung Memorial Hospital and the study's protocol adhered to the tenets of the Declaration of Helsinki.

The enrolled patients were identified according to the causes of their amblyopia. For anisometropic amblyopia, no eye deviation was noted in the cover test. Anisometropia in hyperopia, astigmatism, or myopia was defined as an interocular cycloplegic difference of 2 diopters (D) or more. Amblyopia associated with anisometropia and eye deviation was defined as strabismic anisometropic amblyopia. Patients meeting none of the criteria above were defined as strabismic amblyopia if there was eye deviation or defined as ametropic amblyopia if no eye deviation was found.

Patients were excluded if they had any organic eye disease or a history or evidence of intraocular surgery or trauma or if they were not sufficiently cooperative for an OCT examination. All of the patients underwent a comprehensive ophthalmic examination, including the BCVA, axial length measurement (Optical Biometer AL-Scan; Nidek, Japan), a slit-lamp examination, intraocular pressure assessment (Full Auto Tonometer TX-F; Canon, Japan), extraocular motility assessment, cover-uncover test, and dilated fundoscopy. All of the refraction tests were performed after instilling one drop of 1% cyclopentolate solution and 2 drops of 1% tropicamide solution at 10-minute intervals. Visual acuity testing was performed using the Landolt C chart, following the standard procedure.

**Figure 1:**
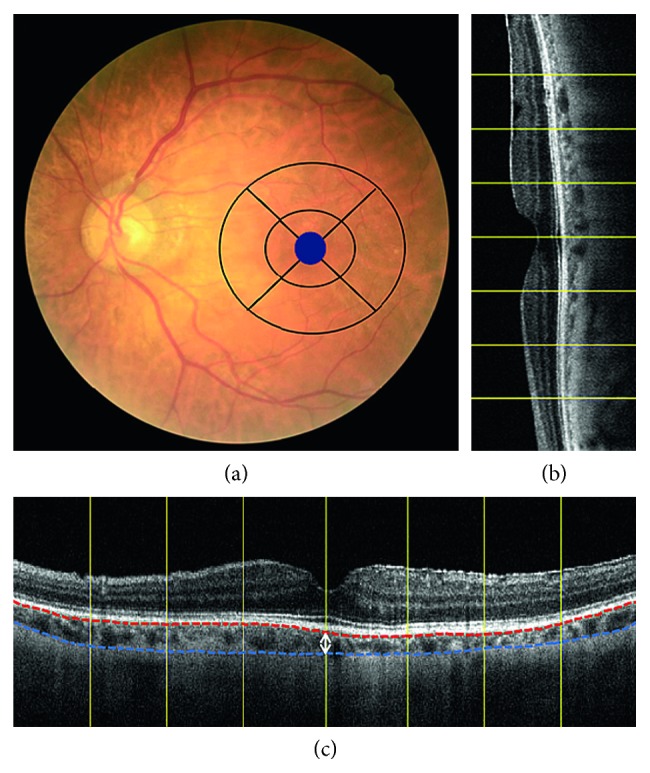
Measurements of the macular and choroidal thicknesses. (a) Macular retinal thickness was measured in three concentric rings, with the central ring corresponding to the fovea (1 mm diameter), the middle ring corresponding to the parafovea (3 mm diameter), and the outer ring corresponding to the perifovea (5 mm diameter). (b, c) The yellow lines indicate the locations of the 1000 *μ*m intervals. The distance between the outer border of the retinal pigment epithelium (red dash line) and the hyperrefractive line behind the large vessel layers of the choroid (blue dash line) was defined as choroidal thickness (white arrow).

Macular and choroidal thicknesses were measured using an SD-OCT system (RTVue, Optovue Inc., Fremont, CA, USA) before the amblyopia treatment. The data for the macular retinal thickness were displayed in 3 concentric circles with diameters of 1 mm (fovea), 3 mm (parafovea), and 5 mm (perifovea). To measure the choroidal thickness, we used the protocols previously described by Wang and associates [10]. Briefly, the choroid was imaged in the “choroidal mode” with the SD-OCT, and its thickness was defined as the distance between the outer border of the retinal pigment epithelium (RPE) and the hyperrefractive line behind the large vessel layers of the choroid, which is presumed to be the choroid-sclera interface (Figure 1). Only the images with a clear choroid-sclera interface were used for analysis. The choroidal thickness was measured manually beneath the fovea using the scale supplied with the system's software at 1000 *μ*m intervals from the fovea to a distance of 3 mm in the nasal, temporal, superior, and inferior directions. The average value of the 14 choroidal thickness readings was recorded as the macular choroidal thickness. The subfoveal choroidal thickness was defined as the choroidal thickness measured at the center of the foveola. Each image was measured by two independent technicians. Data with discrepancies of 20% were reanalyzed by the author (Sherine Jue Ong).

All of the patients received standard treatment including wearing spectacles for refraction correction and occlusion of the dominant fellow eye. The refraction and BCVA were rechecked every 3 months, and the final BCVA value was recorded after 6 months of follow-up. Then, the patients were divided into two groups according to the final BCVA result: those with recovered amblyopia that was defined as a final BCVA 0.8 or better and those with persistent amblyopia who had a final BCVA worse than 0.8.

The statistical analysis was performed using SPSS 20 software (IBM, Armonk, NY, USA). The OCT measurement data were corrected for the axial length-induced ocular magnification using Littmann's formula. In Littmann's formula, which is *t* = *p* × *q* × *s* [11–13]; *t* is the real fundus dimension; *p* is the instrument-dependent magnification constant for the imaging system; *q* is the ocular magnification factor related to the axial length; and *s* is the value obtained using OCT. Logistic regression models were constructed for comparison of each related factor and the OCT data for the groups with persistent and recovered amblyopia. For the children with bilateral amblyopia, the worse eye was used in the analysis. A paired *t*-test was used to compare the amblyopic eye and the dominant fellow eye for each of the patients. Statistical significance was defined as *P* < 0.005.

## 3. Results

A total of 44 patients were enrolled, and the mean age was 7.9 ± 2.9 years (mean ± standard deviation). The mean refractive error was +1.17 ± 4.98 D. Among the 44 patients, 18 patients (40.9%) had a final BCVA worse than 0.8 and were grouped into persistent amblyopia. The other 26 patients (59.1%) had a final BCVA of 0.8 or better and were grouped into recovered amblyopia.  Table 1 summarizes the demographic profiles of these two groups. No significant difference in age, gender, spherical equivalent, or axial length was observed between these two groups. The patients who had severe amblyopia (initial VA of ≤0.3) were significantly predisposed to developing persistent amblyopia (*P* < 0.001). Among the included patients, 20 (45.5%) had anisometropic amblyopia, 12 (27.3%) had strabismic anisometropic amblyopia, 5 (11.3%) had strabismic amblyopia, and 7 (15.9%) had ametropic amblyopia. The rate of strabismic anisometropic amblyopia was significantly higher in the persistent amblyopic group than in the recovered group (44.4% and 15.4%, respectively, *P*=0.049).

The macular and choroidal biometric values of the persistent amblyopic and recovered amblyopic groups are summarized in Table 2. The parafoveal retinal thicknesses, parafoveal retinal volumes, perifoveal retinal thicknesses, and perifoveal retinal volumes were significantly less in the persistent amblyopic group than in the recovered amblyopic group (*P*=0.049, 0.046, 0.043, and  0.048, respectively). A significant difference was also noted in the retinal thicknesses of the parafoveal temporal quadrant (*P*=0.022). However, the differences were no longer existent after data adjustment using Littmann's formula. Based on the quality of the images, the choroidal thickness could be analyzed in only 29 patients (9 in the persistent amblyopic group and 20 in the recovered amblyopic group). There was no significant difference in the choroidal thickness between these two groups.

We further compared the SD-OCT findings between the nondominant amblyopic eyes and the dominant fellow eyes (Table 3). However, there were no significant differences in the macular thickness, macular volume, or choroidal thickness, either before or after correction using Littmann's formula.

## 4. Discussion

In our study, nearly 60% of children achieved a BCVA of 0.8 or better after treatment for amblyopia. Reviewing the previous studies showed that the posttreatment VA was maintained or improved in 47–96.3% of amblyopic patients [14–17]. The risk factors for persistent amblyopia in our study were the type of amblyopia (strabismic anisometropic amblyopia) and the initial severity of the amblyopia (BCVA ≤ 0.3). This finding is compatible with those of other studies reported in the literature [14, 15, 18–20]. Levartovsky et al. reported that the patients with more profound amblyopia had a greater risk of deterioration and developing recurrent amblyopia after discontinuing treatment [14]. Therefore, closer follow-up may be needed for more profoundly amblyopic patients. Some clinicians have reported that patients at a younger age at the beginning of treatment achieved a better outcome [20, 21], although this result was inconsistent with those of other studies [14, 15, 19]. In this study, we did not observe an age difference between the persistent amblyopia and recovered amblyopia groups.

Previous studies have emphasized the differences between the OCT findings in amblyopic eyes compared with those in normal control eyes or in the fellow eyes [9, 22]. The presence of amblyopia was associated with increased foveal thickness, but the origin or significance of this finding is still uncertain. Some clinicians have suggested that differences exist only in some specific types of amblyopia [8, 23–25], but others have reported that no significant differences in the macular structures were found [6, 26]. Bruce et al. found that differences exist between the amblyopic eyes and visually normal eyes but not between the amblyopic eyes and fellow eyes [27]. The abovementioned variations may be due to the lack of control groups, differences in the OCT instruments utilized, variations in the ages of the enrolled subjects, refractive errors, or the types of amblyopia. Considering the effects of the axial lengths and refractive errors on the OCT images, Littmann's formula was used for correction in the present study [11, 12]. The result showed that the corrected macular and choroidal biometric values of the amblyopic eyes and the dominant fellow eyes were not significantly different.

We also found that the corrected macular and choroidal biometric values of the recovered amblyopic eyes and the persistent amblyopic eyes were not significantly different either. This finding is consistent with the results of previous studies [28, 29]. Tugcu et al. studied macular thickness in the persistent amblyopic and resolved amblyopic eyes and did not found significant difference between the two groups [28]. Chen et al. compared the macular thickness of the amblyopic eyes with those of fully corrected previous amblyopic eyes and nonamblyopic controls and found there was no significant difference among the three groups [29]. By contrast, Pang et al. reported that the central macular thickness in myopic anisometropic amblyopia significantly reduced after amblyopia treatment [30]. However, the measurements in their study were not adjusted for axial length and refractive error. To clarify the role of retinal alternation in the amblyopia treatment, further longitudinal and comparative studies are required.

Previous studies have reported the measurements of choroidal thickness in healthy children by different instruments [31–33]. Read et al. reported that the choroid is significantly thinner in children during early childhood than in children of older age groups [31]. The subfoveal choroidal thickness has been reported to be thicker in amblyopic eyes than in control eyes [34]. In our study, the subfoveal choroidal thickness in the amblyopic eye was relatively thinner compared with other studies. Since the choroidal thickness was reported to be greater in the hyperopia than in emmetropia and myopia children [35], one of the children in our study had high myopia of −10.75 D, in which the measured subfoveal choroidal thickness was 143 *μ*m, which might explain the different result. Besides, an increased macular choroidal thickness was found to be related to a better BCVA, less myopia, and a shorter axial length in studies of myopia [10, 36]. Further studies related to the choroidal thickness and amblyopia are worth studying.

There are several limitations to our study. First, there was no normal control group for comparing the macular and choroidal structures. Second, the macular choroidal thickness was measured manually, and new automated software will reduce the bias involved and the time required to obtain measurements of the macular choroidal thickness. Third, a small number of patients were enrolled, precluding subgroup analysis based on the different types of amblyopia. Further long-term studies with larger samples may be needed.

In conclusion, the initial VA and the type of amblyopia play important roles in visual recovery during amblyopic treatment. No difference was found in the macular and choroidal thicknesses between the persistent and recovered amblyopic eyes. There were also no prominent interocular differences in the amblyopic patients. The thickness of the retina and choroid obtained using OCT before treatment might have limited value for predicting the treatment outcome. Further studies with a longer follow-up period are warranted to determine whether retinal or choroidal structures have any effect on the response to amblyopia therapy.

## Figures and Tables

**Table 1:** Demographic profiles of the participants with persistent amblyopia and recovered amblyopia.

	Persistent amblyopia (*n*=18)	Recovered amblyopia (*n*=26)	*P* value
Age			
Mean ± SD, years	7.46 ± 2.13	8.14 ± 3.25	0.436
Range, years	5.0–11.5	5.0–16.0	

Gender			
Male, *n* (%)	12 (66.67)	15 (57.70)	0.549
Female, *n* (%)	6 (33.33)	11 (42.31)	0.552
Severe amblyopia^*∗*^, *n* (%)	15 (83.33)	2 (7.69)	<0.001

Type of amblyopia			
Anisometropic, *n* (%)	6 (33.33)	14 (53.85)	0.111
Strabismic anisometropic, *n* (%)	8 (44.44)	4 (15.38)	0.049
Strabismic, *n* (%)	3 (16.67)	2 (7.69)	0.226
Ametropic, *n* (%)	1 (5.56)	6 (23.08)	0.426

Spherical equivalent^†^			
Mean ± SD, diopters	0.47 ± 5.97	1.61 ± 4.12	0.449
Range, diopters	−12.63 to + 7.25	−10.75 to +8.5	

Axial length^†^			
Mean ± SD, mm	23.05 ± 1.73	22.58 ± 1.33	0.359
Range, mm	20.8–26.34	20.44–26.75	

SD: standard deviation. ^*∗*^Severe amblyopia was defined as visual acuity of <0.3 at the time of entering the study. ^†^The worse eye of the children with bilateral amblyopia was used in the analysis.

**Table 2:** Macular and choroidal thicknesses in persistent amblyopia and recovered amblyopia.

	Persistent amblyopia (*n*=18)	Recovered amblyopia (*n*=26)	*P* value	*P* value^†^
Fovea				
Thickness, *μ*m	238.11 ± 16.27	245.12 ± 28.04	0.343	0.601
Volume, mm^3^	0.19 ± 0.01	0.19 ± 0.02	0.355	0.591

Parafovea				
Thickness, *μ*m	298.83 ± 19.91	310.28 ± 15.13	0.049	0.615
Volume, mm^3^	1.88 ± 0.13	1.95 ± 0.09	0.046	0.892
Temporal thickness, *μ*m	286.72 ± 18.59	300.2 ± 15.71	0.022	0.527
Superior thickness, *μ*m	302.6 ± 26.27	313.88 ± 19.82	0.130	0.496
Nasal thickness, *μ*m	308.11 ± 20.98	317.76 ± 17.2	0.113	0.894
Inferior thickness, *μ*m	298.22 ± 19.29	308.92 ± 16.15	0.065	0.635

Perifovea				
Thickness, *μ*m	283.17 ± 21.09	294.48 ± 11.68	0.043	0.633
Volume, mm^3^	3.56 ± 0.26	3.7 ± 0.15	0.048	0.672
Temporal thickness, *μ*m	276.67 ± 21.56	287.6 ± 14.04	0.061	0.702
Superior thickness, *μ*m	279.17 ± 26.55	292.68 ± 23.42	0.103	0.349
Nasal thickness, *μ*m	299.56 ± 22.72	308.56 ± 13.26	0.121	0.892
Inferior thickness, *μ*m	277.89 ± 21.89	280.16 ± 26.46	0.761	0.734

Choroidal thickness (*n*=29)^*∗*^				
Macular, *μ*m	251 ± 43.45	267.4 ± 42.87	0.343	0.108
Subfoveal, *μ*m	283.78 ± 57.69	295.5 ± 43.87	0.538	0.188

SD: standard deviation. The worse eye of the children with bilateral amblyopia was used in the analysis. Unless otherwise indicated, data are given as the mean ± standard deviation. ^†^*P* value calculated after correcting using Littmann's formula. ^*∗*^Images of good quality, as determined by a clear choroid-sclera interface, were used in the analysis (*n*=29).

**Table 3:** Macular and choroidal thicknesses of the amblyopic eye and the fellow eye.

	Amblyopic eye (*n*=44)	Fellow eye (*n*=44)	*P* value^†^
Fovea			
Thickness, *μ*m	242.19 ± 23.85	237.58 ± 24.47	0.623
Volume, mm^3^	0.19 ± 0.02	0.19 ± 0.02	0.604

Parafovea			
Thickness, *μ*m	305.49 ± 18	304.44 ± 14.63	0.800
Volume, mm^3^	1.92 ± 0.11	1.91 ± 0.09	0.487
Temporal thickness, *μ*m	294.56 ± 18.06	295.23 ± 15.12	0.407
Superior thickness, *μ*m	309.16 ± 23.14	307.98 ± 19.45	0.951
Nasal thickness, *μ*m	313.72 ± 19.25	311.74 ± 16.45	0.994
Inferior thickness, *μ*m	304.44 ± 18.11	302.49 ± 15.53	0.999

Perifovea			
Thickness, *μ*m	289.74 ± 17.03	287.14 ± 14.07	0.748
Volume, mm^3^	3.64 ± 0.21	3.56 ± 0.31	0.219
Temporal thickness, *μ*m	283.02 ± 18.18	279.72 ± 14.06	0.785
Superior thickness, *μ*m	287.02 ± 25.37	288.02 ± 14.95	0.443
Nasal thickness, *μ*m	304.79 ± 18.15	303.09 ± 15.22	0.925
Inferior thickness, *μ*m	279.21 ± 24.4	275.6 ± 26.48	0.142

Choroidal thickness (*n*=29)^*∗*^			
Macular, *μ*m	262.31 ± 42.97	260.3 ± 36.38	0.771
Subfoveal, *μ*m	297.83 ± 47.83	289.59 ± 43.2	0.924

SD: standard deviation. Unless otherwise indicated, data are given as the mean ± standard deviation. ^†^*P* value calculated after correcting using Littmann's formula. ^*∗*^Images of good quality, as determined by a clear choroid-sclera interface, were used in the analysis (*n*=29). In children with bilateral amblyopia, the worse eye was considered to be the amblyopic eye and the other eye was considered to be the dominant fellow eye.

## Data Availability

The data used to support the findings of this study were provided by Chang Gung Memorial Hospital under license and so cannot be made freely available. Access to these data will be considered by the author upon request, with permission from Chang Gung Memorial Hospital.
